# The role of fibroblast growth factor receptor 2 (FGFR2) in differentiation of bovine spermatogonial stem cells (SCC)

**Published:** 2016-06-15

**Authors:** Vahid Akbarinejad, Parviz Tajik, Mansoureh Movahedin, Reza Youssefi

**Affiliations:** 1 *Department of Theriogenology, Faculty of Veterinary Medicine, University of Tehran, Tehran, Iran; *; 2 *Theriogenology Association, Faculty of Veterinary Medicine, University of Tehran, Tehran, Iran; *; 3 *Department of Anatomical Sciences, Faculty of Medical Sciences, Tarbiat Modares University, Tehran, Iran.*

**Keywords:** Bovine, Differentiation, Fibroblast growth factor receptors, Spermatogonial stem cells

## Abstract

The receptors 1 and 2 of fibroblast growth factor (FGFR1 and FGFR2, respectively) have been observed in all types of testicular cells. Culture on extracellular matrix (ECM) has been observed to lead to initiation of differentiation in spermatogonial stem cells (SSCs). The present study was carried out to investigate whether FGFR1 and FGFR2 play a role in SSCs differentiation. Following isolation, bovine testicular cells were cultured on ECM-coated or uncoated (control) plates for 12 days. The gene expression of THY1, cKIT, FGFR1 and FGFR2 was evaluated using quantitative real-time polymerase chain reaction (PCR). Results related to the gene expression of markers of with undifferentiated (THY1) and differentiated (cKIT) spermatogonia implicated stimulation of self-renewal and differentiation in cells cultured on ECM-coated and uncoated plates, respectively (*p* < 0.05). Concomitantly, the expression of FGFR2 increased during culture in the ECM group (*p *< 0.05), whereas it did not change in the control group (*p* > 0.05). As a result, the gene expression of FGFR2 was greater in the ECM than control group (*p* < 0.05). Nevertheless, FGFR1 expression did not change during culture in the control and ECM groups (*p* > 0.05). In conclusion, the present study revealed the potential role of FGFR2 in differentiation of SSCs during culture on ECM.

## Introduction

Spermatogenesis is an intricate and tightly-regulated process of cell proliferation and differentiation leading to production of mature spermatozoa from spermatogonial stem cells (SSCs).^1^ Maintenance of spermatogenesis depends on capability of SSCs to both self-renewal and differentiation properties.^1^ Coordination of spermatogenesis, and the balance between SSCs self-renewal and differentiation is regulated by growth factors and their receptors.^1^ The niche consists of different somatic cells surrounding SSCs and governing the spermatogenesis by producing various growth factors.^1^ One of the growth factors which plays a role in proliferation SSCs is fibroblast growth factor.^2^^,^^3^

Extracellular matrix (ECM) is the other part of niche, made up of a complex network of macromolecules with different structural and functional properties modulating the function and development of cells.^4^^,^^5^ *In vitro* culture on ECM provides an environment partly similar to the normal tissue,^6^^-^^8^ which allows the initiation of SSCs differentiation.^9^^-^^11^

Fibroblast growth factor receptors (FGFRs) are evolutionarily conserved trans-membrane proteins that are composed of an extracellular ligand-binding domain, a transmembrane region and a cytoplasmic portion containing the catalytic protein tyrosine kinase domain.^12^^,^^13^ Conventional gene knockout of either FGFR1 or FGFR2 results in an early death in utero, suggesting the vital role of these receptors during embryonic development.^14^^,^^15^ Li *et al*. demonstrated the expression of FGFR1 and FGFR2 in all types of testicular cells in mice.^16^ However, the role of FGFR1 and FGFR2 has not investigated in bovine SSCs development. Nevertheless, FGFR1 and FGFR2 have been reported as the main receptors of mediating fibroblast growth factors (FGFs) action in bovine corpus luteum.^17^ The FGFRs have been observed to be expressed in cumulus cells as well as oocytes and play an important role in bovine oocyte maturation.^18^ Further, FGFRs have been detected in bovine embryos and it was observed that FGFRs contribute to interferon t expression.^19^ The present study was conducted to elucidate whether FGFR1 and FGFR2 contribute to the differentiating effect of ECM on SSCs.

## Materials and Methods

**Animals and testicular biopsy. **Animal Ethics Committee at University of Tehran, Tehran, Iran, approved the present study in terms of animal welfare and ethics. To obtain testicular tissue, Holstein calves (aged 3 to 5 months) were subjected to testicular biopsy as previously described.^11^ In brief, testicular biopsy was performed under sedation with xylazine (0.2 mg kg^-1^; Alfasan, Woerden, Holland) and local anesthesia with lidocaine (Aburaihan Pharma Co. Tehran, Iran). Following incision, the testicular tissue was obtained and placed into a 15 mL tube containing Dulbecco's Modified Eagle's medium (DMEM; Gibco, Carlsbad USA) with 10% fetal bovine serum (FBS, Sigma-Aldrich, St. Louis, USA) and antibiotics (100 IU per mL penicillin and 100 μg mL^-1^ streptomycin; Gibco). The specimen was subsequently transferred on ice to the laboratory within 2 hr. 

**Cell isolation. **Cell isolation was implemented using a two-step enzymatic isolation procedure, as previously described.^11^ In brief, the testicular tissue was washed three times in DMEM containing antibiotics and was minced into small pieces by a sterile scissor. The minced testicular tissue was incubated in DMEM containing 1 mg mL^-1^ collagenase (Sigma-Aldrich, St. Louis, USA), 1 mg mL^-1^ hyaluronidase (Sigma-Aldrich), 1 mg mL^-1^ trypsin (Sigma-Aldrich) and 5 µg mL^-1^ DNase (Fermentas, St. Leon-Rot, Germany) at 37 ˚C in a shaker incubator with 80 cycles per min for approximately 60 min. The digested testicular tissue was washed three times with DMEM and the supernatant was disposed after each washing, leading to isolation of seminiferous tubules. During the second step of enzymatic digestion, the seminiferous tubules were incubated at 37 ˚C in DMEM containing 1 mg mL^-1^ collagenase, 1 mg mL^-1^ hyaluronidase and 5 µg mL^-1^ DNase until disintegration of the seminiferous tubules and separation of the constituent cells. Individual cells were isolated from the remaining tubule fragments by centrifugation at 30 *g* for 2 min. Following filtration through 77 and 55 µm nylon filters, the cells were pelleted. The pellet was re-suspended in the DMEM containing antibiotics and 10% knock-out serum replacement (KSR; Gibco). 

**Cell culture. **Wells used for the control group were uncoated. Wells used for the extracellular matrix (ECM) group were coated with ECM gel (Sigma-Aldrich). The ECM gel was prepared from Engelbreth-Holm-Swarm (EHS) mouse sarcoma and was composed of laminin as the major component, collagen type IV, heparan sulfate proteo-glycan, entactin and other minor components. Six-well plates were coated with extracellular matrix (ECM) gel (Sigma-Aldrich) as the manufacturer indicated. Cells were seeded at concentrations of 1,000,000 cells per well containing DMEM with antibiotics and 10% KSR.^11^ The plates were incubated at 37 ˚C in a humidified atmosphere with 5% CO_2_. The medium was replaced with fresh one every three days.

**RNA isolation and quantitative real-time PCR (qRT-PCR). **Following trypsinization of the cultured cells (n = 3; cell populations from different calves), the isolated cells were subjected to total RNA extraction using Trizol reagent (Fermentas). The extracted RNA was treated with DNase (Fermentas) to eliminate DNA contamination. The concentration of RNA was determined by UV spectro-photometry (Eppendorf, Hamburg, Germany). The cDNAs were synthesized from 500 ng of RNA by oligo (dT) primers using RevertAid™ First Strand cDNA synthesis kit (Fermentas). Primers for genes of interest are shown in Table 1. PCRs were performed using Master Mix and SYBR Green I (Fermentas) in a thermal cycler (model StepOne™; Applied Biosystems, Foster City, USA). The PCR program started with an initial melting cycle for 5 min at 95 ˚C to activate the polymerase, followed by 40 cycles of melting (30 sec at 95 ˚C), annealing (30 sec at 58 ˚C) and extension (30 sec at 72 ˚C). The quality of the PCR reactions was confirmed by melting curve analyses. For each sample, the reference gene (β-ACTIN) and target gene were amplified in the same run. The target genes were normalized to the reference gene. The mean target gene threshold cycle (Ct) and mean exogenous control (β-ACTIN) Ct for each sample were calculated from duplicate wells. The target gene threshold cycle (Ct) of the control was subtracted from the Ct of target gene, resulting in ∆Ct. In each experiment, the Ct of time-point 0 sample was considered as calibrator. Subsequently, the ∆Ct of sample was then subtracted from the ∆Ct of calibrator, resulting in the ∆∆Ct, which was used for calculation of the relative amounts of target gene expression for each sample.^20^

**Statistical analysis. **Data related to gene expression were analyzed using MIXED procedure. In addition, LSMEANS statement was used to perform multiple comparisons. All analyses were conducted in SAS (version 9.2, SAS Institute Inc., Cary, USA). Data are presented as mean ± SD. Differences with *p* < 0.05 were considered significant. 

## Results

**Fig. 1:**
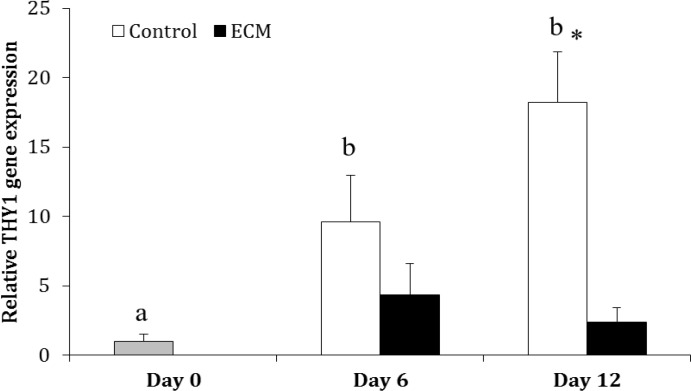
Relative gene expression of THY1 in the control and ECM groups (n = 3) on Days 0, 6 and 12.

^ab^ Different letters indicate significant difference within groups between different time-points (*p* < 0.05). Asterisk indicates significant difference between two experimental groups at the specified time-point (*p* < 0.05).

In the control group, the gene expression of THY1 was greater on Days 6 (9.59 ± 3.38 fold) and 12 (18.23 ± 3.62 fold) than Day 0 (*p* < 0.001), but it was not different between Days 6 and 12 (*p* > 0.05). In ECM group, the expression of THY1 did not differ among days of culture (*p* > 0.05). The expression of THY1 was not different between two experimental groups on Day 6 (*p* > 0.05); however, it was higher in the control than ECM group on Day 12 (*p* < 0.0001; Fig. 1).

In the control group, the expression of cKIT on Days 6 and 12 was 92.00% and 93.00% respectively lower than that on Day 0 (*p* < 0.0001), howerer, it was not different between Days 6 and 12 (*p* > 0.05). In ECM group, the expression of cKIT on Days 6 and 12 was 78.00% and 69.00% respectively lesser than that on Day 0 (*p* < 0.0001); however, it did not differ between Days 6 and 12 (*p* > 0.05).

**Table 1:** Primer sequences used for qRT-PCR

**Gene**	**Forward primer (5´-3´)**	**Reverse primer (5´-3´)**
**β-ACTIN**	TCG CCC GAG TCC ACA CAG	ACC TCA ACC CGC TCC CAA G
**THY1**	TTC ATC TCC TTG TGA CGG GTT	GCA GAG GTG AGG GAA TGG C
**cKIT**	TAC CAA CCA AGG CAG ACA A	CTT TGA GGC AAG GAA CGC
**FGFR1**	ACTGCTGGAGTTAATACCACCG	GCAGAGTGATGGGAGAGTCC
**FGFR2**	CACCACGGACAAAGAAATTG-3'	ATGCAGAGTGAAAGGATATCCC

**Fig. 2:**
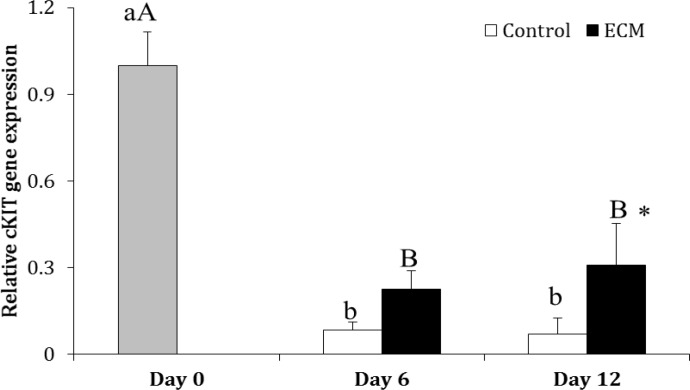
Relative gene expression of cKIT in the control and ECM groups (n = 3) on Days 0, 6 and 12.

^ab, AB^ Different letters indicate significant difference within groups between different time-points (*p* < 0.05). Asterisk indicate significant difference between two experimental groups at the specified time-point (*p* < 0.05).

**Fig. 3:**
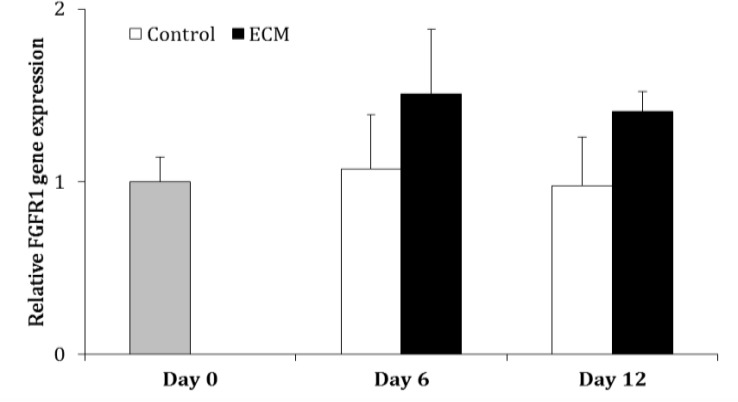
Relative gene expression of FGFR1 in the control and ECM groups (n = 3) on Days 0, 6 and 12. There is no significant difference between the experimental groups

The expression of cKIT in ECM group did not differ between two groups on Day 6 (*p* > 0.05), but it was higher than in the ECM than control group on Day 12 (*p* < 0.05; Fig. 2). The gene expression of FGFR1 did not change during culture in the control and ECM groups (*p* > 0.05). In addition the expression of FGFR1 was not different between two groups on Days 6 and 12 (*p* > 0.05; Fig. 3).

**Fig. 4:**
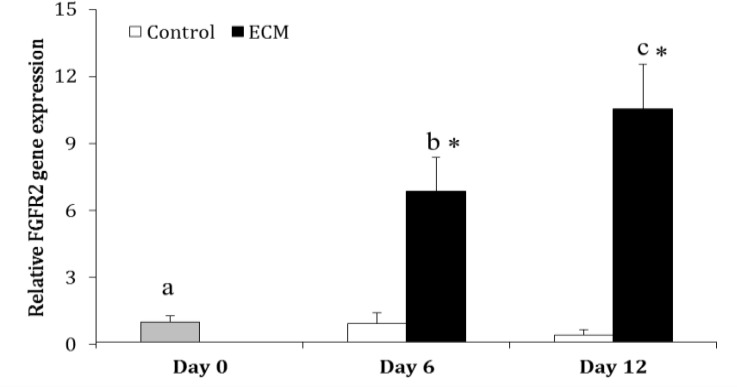
Relative gene expression of FGFR2 in the control and ECM groups (n = 3) on Days 0, 6 and 12.

^abc^ Different letters indicate significant difference within groups between different time-points (*p* < 0.05). Asterisks indicate significant difference between two experimental groups at the specified time-point (*p* < 0.05).

In the control group, the expression of FGFR2 did not change over the culture (*p* > 0.05). Whereas in ECM group, the expression of FGFR2 was higher on Days 6 (6.85 ± 1.52 fold) and 12 (10.55 ± 1.97 fold) as compared with Day 0 (*p* < 0.0001); moreover, it was greater on Day 12 than 6 (*p* = 0.0006). The expression of FGFR2 was higher in the ECM than control group on Days 6 and 12 (*p* < 0.001; Fig. 4).

## Discussion

The THY1 is considered as a conserved marker for undifferentiated spermatogonia including SSCs in a broad range of mammals.^7^^,^^21^^-^^24^ Increase in the expression of THY1 following conventional culture in the present study is consistent with the findings of the study carried out by Akbarinejad *et al*. and Nasiri *et al*.^11^^,^^25^ In this regard, Oatley *et al*. have also reported increase in the number of bovine germ cells during conventional culture.^26^ On the other hand, cKIT has been identified as a marker for differentiated spermatogonia, and SSCs are believed to be negative for cKIT.^27^^,^^28^ Therefore, culture on ECM-coated plates potentiated differentiation in spermatogonia as the higher expression of cKIT in cells exposed to ECM indicated, which agrees with findings of the study by Akbarinejad *et al*.^11^ Likewise, Lee *et al*. found evidence for germ cell differentiation following culture of testicular cells with ECM, the phenomenon which was not observed in cells cultured on uncoated plates.^10^ The same observation was indicated when ECM was used for the culture of human testicular germ cells.^9^

The expression of FGFR1 did not change over the course of culture, and in turn, did not differ between cells cultured on ECM and plastic. This observation suggests that FGFR1 might not play a role in SSCs self-renewal or differentiation. Li *et al*. has also indicated that FGFR1 was not essential for spermatogenesis in mice though it was expressed in different types of testicular cells.^16^

Nevertheless, the expression of FGFR2 significantly increased following culture of ECM and was greater in cells culture on ECM-coated plates than those cultured on uncoated plates. This phenomenon indicates that FGFR2 probably contribute to SSCs differentiation. Likewise, FGFR2 has been identified to be involved in osteogenic differentiation of murine mesenchymal stem cells.^29^ Moreover, Garcia *et al*. demonstrated that FGFR2 signaling is required for derivation of lens fiber cells out of the cell cycle during the terminal differentiation as well as for normal elongation of primary lens fiber cells and to the survival of lens epithelial cells.^30^ Zhang *et al*. has also reported the essential role of FGFR2 in proliferation and differentiation of corneal epithelium during embryonic development.^31^ Evaluating the expression of FGF2 in testicular cells cultured on ECM-coated and uncoated plates, Akbarinejad *et al*. found no alteration in FGF2 expression over the course of culture.^11^ As a result, it could be surmised that even though ECM do not impact the expression FGF2, it could alter the expression of its receptor, thereby regulating SSCs fate. Further studies are warranted to reveal the role of FGF2 and its receptors in regulation of SSCs function.

In conclusion, the results of the present study provided evidence for differentiation of SSCs following culture on ECM. In addition, the present study indicated the potential contribution of FGFR2 in differentiation of SSCs in response to ECM, which, to our knowledge, is the first report in this regard.
